# Clinician Perspectives on Implementing Video Visits in Home-Based Palliative Care

**DOI:** 10.1089/pmr.2020.0074

**Published:** 2020-10-06

**Authors:** Thearis A. Osuji, Mayra Macias, Carmit McMullen, Eric Haupt, Brian Mittman, Richard A. Mularski, Susan E. Wang, Henry Werch, Huong Q. Nguyen

**Affiliations:** ^1^ Kaiser Permanente Southern California, Department of Research & Evaluation, Pasadena, California, USA.; ^2^ Kaiser Permanente Northwest, Center for Health Research, Portland, Oregon, USA.; ^3^ Kaiser Permanente Southern California, West Los Angeles Medical Center, Los Angeles, California, USA.; ^4^ Kaiser Permanente Northwest, Member-Caregiver, Stakeholder Advisory Committee, Portland, Oregon, USA.

**Keywords:** adoption, clinicians, implementation, palliative care, telehealth, video consultations

## Abstract

***Background:*** Despite the increasing use and acceptance of technology in health care, there is limited evidence on the usefulness and appropriate use of telehealth in home-based palliative care (HBPC). As part of the process evaluation of a pragmatic trial of video visits in HBPC, we assessed clinician experience with video visit implementation.

***Methods:*** We assessed clinicians' experiences with and perception of the usefulness and appropriateness of video visits using anonymous surveys and brief qualitative interviews with a subset of survey participants. Qualitative analyses were guided by sociotechnical frameworks that emphasize technology's “value proposition” for its end users as being key to adoption.

***Results:*** Clinicians (36 physicians and 48 registered nurses) generally had favorable attitudes toward video visits and telehealth. Respondents felt confident in the skills needed to make their role in video visits successful. Clinicians were neutral on whether video visits were useful for their practice or enhanced the patient–caregiver experience. Clinicians found video visits to be most appropriate for follow-up care (as opposed to start of care). The interviews yielded two themes that complemented the survey findings: (1) factors enhancing the value proposition (positive responses from patients and families and convenience) and (2) factors diminishing the value proposition (issues related to the technology and scheduling).

***Discussion:*** Our findings provide insights into clinicians' experiences with implementing remote video physician consultations, facilitated by a nurse in the patient's home in the pre-COVID-19 era. Clinician views about video visits may have shifted with the pandemic, which occurred after our data collection was complete.

Clinical Trials Registration No. NCT#03694431.

## Introduction

Advances in technology such as the use of telehealth or video consultation (video visits) have made it feasible for clinicians to provide clinical care and support in the home setting.^1^ Video visits allow patients to have face-to-face interaction with providers, enable providers to observe patients and conduct visits, while at the same time lessening travel burden.^1–7^ Several studies indicate that clinician acceptance, driven by its relative value, is the most important factor in the uptake and utilization of video visits.^5–9^ Despite the increasing use and acceptance^7^ of video visits between clinic-based physicians with patients in their homes,^10^ there is limited information on how physicians and nurses experience the use of facilitated video visits with patients and family caregivers in home-based palliative care (HBPC).

This report is a substudy of a larger comparative effectiveness trial (HomePal) that compared a standard HBPC model with a tech-supported model that included use of nurse to physician video consultation.^11^ Standard HBPC typically included separate home visits by physicians and nurses. The tech-supported model relied on the nurse who was in the patient's home to facilitate a synchronous video consultation between the patient and family with the remote physician; this approach to video visit use was intended to minimize disparities in access to technology by patients and families. We expected that early video access to the physician for discussion and concurrence on the treatment plan would build trust and confidence with the patient/caregivers, potentially reducing the need for an in-home physician visit. Physicians had the discretion of conducting home visits as needed in the tech-supported model. Half of the teams were randomized to implement the standard HBPC model and the other half, the tech-supported model.

The aim of this analysis was to assess the usefulness and appropriateness (i.e., value proposition) of video visits from the perspective of HBPC physicians and nurses, anchored within the NASSS framework^12,13^ (nonadoption and abandonment of technologies by individuals and the challenges to scale-up, spread, and sustainability of such technologies in health and care organizations).

## Methods

We conducted a cross-sectional anonymous survey of HBPC clinicians across 14 study sites to assess the usefulness and appropriateness of video visits and attitudes toward telehealth. The survey was administered approximately eight months after the trial started enrolling participants. The survey was approved by our institutional review board as part of the main study.^11^ All HBPC clinicians (physicians, *n* = 83; full- and part-time registered nurse case managers, *n* = 105) were invited to participate in the survey through e-mail and participation was strictly voluntary. Respondents completed web (REDCap) or paper surveys.

A 26-item physician and a 20-item nurse surveys were developed based on a review of the literature^14–19^ and underlying conceptual framework of relevant study-specific domains: quality of physician–patient communication, perceived use and usefulness, satisfaction with the current program, perceptions of telehealth, and perceived effects on patients and caregivers. All questions used a 5-point Likert scale (1 = strongly disagree to 5 = strongly agree) with the exception of one question on perceived effects of video visits on patients–family caregivers (−5 = very negatively to 0 = neutral to 5 = very positively).

To supplement the survey data and further assess clinician perspectives and overall experience on the use of video visits, clinicians were invited to participate in 10–15 minutes semistructured interviews over the phone or in-person upon completing the survey. Three qualitative interviewers (T.O., M.M., and H.N.) used a semistructured guide to probe the clinicians' experiences with video visit implementation, barriers and facilitators, and changes and recommendations. A note-taking template was used to systematically record the interview data.

## Analysis

We computed descriptive statistics to summarize survey results. Interview notes were imported to NVivo for coding and analysis by T.O. and M.M. and were reviewed with C.M. to further validate the thematic coding.^20^ In this article, we report on the analysis of one theme, which was heavily represented in the data: the value proposition of video visits to clinicians.

## Results

### Survey

A total of 84 clinicians responded to the survey, which included 36 physicians (45%) and 48 (49%) nurse case managers. At least one physician and one nurse from each of the 14 sites participated in the survey. All physician respondents reported having conducted video visits and half of the nurses reported that they conducted video visits as expected per the HomePal study design (Table 1).

**Table 1.** Clinician Survey Results

Sample description^a^	Physicians (n = 36)	Nurses (n = 48)
Years in current role		
<2 years	5 (14%)	10 (21%)
2–10 years	19 (53%)	26 (54%)
≥10 years	11 (31%)	12 (25%)
Missing	1 (3%)	0 (0%)
Use of video visits		
Conducted video visits (yes)	36 (100%)	27 (56%)
Types of visits conducted using video		
Start-of-care	16 (44%)^b^	7 (26%)
Follow-up	28 (76%)	27 (100%)
Types of HomePal visits appropriate for video (1 = strongly disagree to 5 = strongly agree)		
Start-of-care	2.9 (1.5)	3.4 (1.5)
Follow-up: change in condition	4.1 (1.0)	4.3 (1.1)
Follow-up: resumption of care	4.2 (1.0)	3.8 (1.1)
Follow-up: recertification	4.1 (1.0)	3.8 (1.1)
Follow-up: transition from HomePal	3.8 (1.2)	3.1 (1.3)
Follow-up: other	3.4 (1.0)	3.4 (1.0)
Comfort with conducting video visits (1 = strongly disagree to 5 = strongly agree)		
I have the skills that I need to make my role in video visits successful	4.2 (0.9)	4.6 (0.7)
Competence in doing the following in context of video visit^b^		
Assessing physical, emotional, spiritual, and social needs	3.5 (1.2)	
Expressing empathy	3.9 (1.0)	
Discussing treatment options	4.0 (1.0)	
Discussing transition to hospice	3.8 (1.2)	
Understanding patient's goals for end of life	3.9 (0.9)	
Dealing with conflict between team members	3.2 (1.1)	
Effects of video visits on clinical practice (1 = strongly disagree to 5 = strongly agree)		
Using video visits increases my productivity	3.6 (1.1)	3.0 (1.3)
Using video visits makes it easier to do my job	3.5 (1.3)	3.2 (1.3)
Attitudes toward video visits and telehealth (1 = strongly disagree to 5 = strongly agree)		
Overall, I find that using the technology hinders the care experience	2.4 (1.2)	3.0 (1.3)
I find video visits to be a useful addition to HomePal services	4.0 (1.0)	4.0 (1.2)
Overall, I am satisfied with HBPC video visits	3.4 (1.1)	3.8 (1.1)
I prefer to provide care face-to-face rather than using any form of telehealth technology	3.1 (1.2)	3.2 (1.1)
Telehealth will be a standard way of health care delivery in the future	4.0 (1.2)	4.1 (1.1)
Perceived impact on patients and families (−5 = very negatively to 0 = neutral to 5 = very positively)		
Perceived effect of video visits on patients/families	2.3 (1.7)	2.0 (2.6)

^a^ Data are presented as *n* (%) or mean (standard deviation).

^b^ Item asked of physicians only.

HBPC, home-based palliative care.

#### Use of video visits

Video visits were used primarily for follow-up visits as opposed to admission/start-of-care. Of the 36 physicians who reported having conducted a video visit, 16 (44%) reporting having conducted video visits for admission/start-of-care, whereas 28 (76%) reported conducing video visits at follow-up. Findings among nurses were similar. We also asked physicians which types of visits were most appropriate for video; the results were consistent with physicians indicating a preference for use of video for follow-up care.

#### Comfort with conducting video visits

Both physicians (4.2 [0.9]) and nurses (4.6 [0.7]) agreed that they had the skills needed to be successful in their respective roles during video visits. Physicians felt competent in their ability to perform key activities in the context of a video visit: discussing treatment options, expressing empathy, understanding patient goals for end of life, discussing transition to hospice, assessing physical, emotional, spiritual, and social needs, and dealing with conflict between team members.

#### Effects of video visits on clinical practice

In general, clinicians were neutral about video visits being useful for their practice: using video visits increases my productivity (physicians: 3.6 [1.1]; nurses: 3.0 [1.3]); using video visits makes it easier to do my job (physicians: 3.5 [1.3]; nurses: 3.2 [1.3]).

#### Attitudes toward video visits and telehealth

Based on responses rated on a 5-point scale (1 = strongly disagree to 5 = agree), clinicians had generally positive attitudes toward video visits and telehealth. They reported that video visits were a useful addition to HBPC services, that telehealth will be a standard way of health care delivery in the future, and overall satisfaction with the video visits. Physicians and nurses generally disagreed that technology hinders the care experience. Attitudes were more neutral with regard to preference for in-person care among both physicians (3.1 [1.2]) and nurses (3.2 [1.1]).

#### Perceived impact on patients and families

We asked clinicians to rate the extent to which they believe video visits has had a negative (−5 to −1), neutral (0), or positive (+1 to +5) effect on patients and families. Both physicians (2.3 [1.7]) and nurses (2.0 [2.6]) had neutral to positive response to the effect of video visits on patients and families, although the distribution was wide.

#### Debrief interview

Seven physicians and seven nurses provided additional feedback on their experience with video visits. Two overarching themes emerged from these interviews (Table 2):

**Table 2.** Clinician Interview Results

Theme	Findings	Illustrative comments (paraphrased)
Factors enhancing value proposition for HBPC clinicians	Patients and family members had positive reactions to video visits Video visits offer convenience. Most physicians suggested that the video visits created efficiencies in their work (e.g., convenience of not having to travel), which allows them to see more patients or see HBPC patients more often. Some clinical issues such as medication management, symptom exacerbation, and triage were managed effectively with video visits.	Video visits have been great; all our patients and family enjoy them as it gives them additional contact with me. I haven't received any negative feedback from patients/families. (MD) Initially, I was like oh my god why was I chosen for this, its gonna take all my time and I won't be able to get things done, but then… now I love it! My perception changed as I did more visits, now I want all my patients to have a video visit. (RN) When I did my first follow-up visit with a stable patient, it was great. I saved a car trip and was able to assess the patient's status. (MD) Clinically, the VV has helped to assess how the patient looks, their disease progression to plan for any transitions. In the past, the reports might be delayed for up to two weeks since we depend on the report out from IDG. VV help accelerate communications around emergent issues. I feel less stressed and anxious about being in the dark regarding the patients' status and family is also more aware of the greater involvement by the MDs (RN)
Factors diminishing the value proposition for HBPC clinicians	When technology is hard to use (due to connectivity and quality issues), it greatly diminishes the value of video visits. Physicians and nurses noted that video visits place additional burden on nurses, who set up and facilitate video visits during their home visits. Scheduling is an ever-present challenge. Clinicians reported that some clinical issues, such as visualizing skin problems, were not easy to manage by video.	Half of the time we have connectivity issues. Sound is also poor. Most of our patients are elderly and hard of hearing, which makes it hard even when we have the volume all the way up. (RN) Connectivity is not reliable or high quality. Really need to ensure stable Internet connections at both ends for this to work. When I'm in my car, I can activate my wi-fi hot spot. In some situations, we've been able to use the patient's home wi-fi for a better signal. (MD) We have also tried to use video visits for wound assessments. Again, the quality isn't great as I'm not able to see drainage, color *etc.* (MD) I wouldn't recommend video to someone. The video visits make the visits much longer, sometimes it adds like 30 or 45 minutes. (RN) This [video visits] also adds extra work, because I take my notes on paper during the visit then I have to document all my notes that I take during the visit. This can then delay me for the rest of the day and add time. (RN) I worry about the additional time the video visits add to the RN's schedule. (MD) It just takes a bit coordination with MD. Trying to fit video visits into already busy MD schedule can be a challenge. They just have so many things going on. I usually text them a reminder the morning of the visit. We are still working to figure out what works best for us [the RN and MD]. (RN)

HBPC, home-based palliative care; IDG, interdisciplinary group; V V, video visit.

#### Factors enhancing the value proposition for video visits

Clinicians shared that patients and families responded positively to video visits. They also noted that video visits offer convenience, especially for physicians.

#### Factors diminishing the value proposition video visits

Clinicians noted that issues related to the technology (e.g., connectivity and quality) and scheduling were substantial barriers to conducting video visits. Video visits placed additional burden on the nurses who facilitated the encounters.

## Discussion

The survey and interviews provided complementary findings and insights into clinicians' experiences with implementing video visits as part of the HomePal trial. Clinicians experienced both benefits and challenges of delivering care through the video platform. Survey results showed that video visits were primarily used for follow-up visits and that physicians felt competent providing some services through video. This finding may be explained by the scheduling and coordination challenges experienced with video visits. Both survey and interview findings suggest that video visits offer certain conveniences for some clinicians. The interview findings clarified that video visits placed additional burden on the nurse case managers who facilitated the video visits. Survey results indicate that clinicians have generally positive attitudes toward video visits and telehealth. The interview findings further highlight that clinician enthusiasm may be tempered by technology issues.

Our study has limitations that are important to consider when interpreting the results. The data are cross-sectional, and as such the findings represent a snapshot of clinicians' views of and experiences with video visits. The survey and interviews included a convenience, volunteer sample of clinicians participating in the trial. Although clinicians represented a range of perspectives from different study sites, respondents may not be representative of all HBPC clinicians. Finally, this study was focused on only one of many use cases for video visits and was conducted before the COVID-19 pandemic.

Findings from this report further reinforce the lessons learned from the HomePal trial that are reported elsewhere.^21^ Given the unprecedented adoption of virtual care in response to the pandemic, efforts should focus on ensuring more reliable connectivity so clinicians can focus on providing care, not technical troubleshooting. In addition, optimizing physician and nurse schedules through different arrangements, including assigning a pool of dedicated physicians to provide video consultations on demand, may address the flexibility nurses need in the home care setting. Addressing these two issues are critical for streamlining the video consultations for physicians, nurses, patients, and families.

## Abbreviation Used

HBPC: home-based palliative care
